# Genome sequencing identifies a homozygous inversion disrupting *QDPR* as a cause for dihydropteridine reductase deficiency

**DOI:** 10.1002/mgg3.1154

**Published:** 2020-02-05

**Authors:** Hardo Lilleväli, Sander Pajusalu, Monica H. Wojcik, Julia Goodrich, Ryan L. Collins, Ülle Murumets, Pille Tammur, Nenad Blau, Kersti Lilleväli, Katrin Õunap

**Affiliations:** ^1^ Department of Clinical Genetics, United Laboratories Tartu University Hospital Tartu Estonia; ^2^ Institute of Biomedicine and Translational Medicine University of Tartu Tartu Estonia; ^3^ Department of Genetics Yale University School of Medicine New Haven CT USA; ^4^ Department of Clinical Genetics Institute of Clinical Medicine University of Tartu Tartu Estonia; ^5^ The Broad Institute of MIT and Harvard Cambridge MA USA; ^6^ Divisions of Genetics and Genomics and Newborn Medicine Department of Pediatrics Children’s Hospital Boston MA USA; ^7^ Program in Bioinformatics and Integrative Genomics Division of Medical Sciences Harvard Medical School Boston MA USA; ^8^ Dietmar‐Hopp Metabolic Center University of Heidelberg Heidelberg Germany; ^9^ Division of Metabolism University Children’s Hospital Zürich Switzerland

**Keywords:** dihydropteridine reductase deficiency, genome sequencing, inversion, QDPR gene, tetrahydrobiopterin deficiencies

## Abstract

**Background:**

Dihydropteridine reductase (DHPR) is one of the key enzymes for maintaining in the organism the supply of tetrahydrobiopterin (BH_4_), an essential cofactor for aromatic amino acid hydroxylases. Its dysfunction causes the condition of hyperphenylalaninemia together with the lack of neurotransmitters.

**Methods:**

We report a patient with biochemically diagnosed DHPR deficiency, with extensive molecular investigations undertaken to detect variations in quinoid dihydropteridine reductase (QDPR) gene. Sanger sequencing of QDPR coding regions, exome sequencing, QDPR mRNA PCR, and karyotyping were followed by trio genome sequencing.

**Results:**

Short‐read genome sequencing revealed a homozygous 9‐Mb inversion disrupting QDPR. Structural variant breakpoints in chromosome 4 were located to intron 2 of QDPR at Chr4(GRCh38):g.17505522 and in intron 8 of the ACOX3 gene, Chr4(GRCh38):g.8398067). Both nonrelated parents carried the variant in heterozygous state. The inversion was not present in gnomAD structural variant database.

**Conclusion:**

Identification of the exact breakpoints now allows further straightforward molecular genetic testing of potential carriers of the inversion. This study extends the pathogenic variant spectrum of DHPR deficiency and highlights the role of structural variants in recessive metabolic disorders. To our knowledge, this is the first report on a large, canonical (rather than complex) homozygous pathogenic inversion detected by genome sequencing.

## Graphical abstract

We present a case in whom genome sequencing revealed a homozygous 9‐Mb inversion disrupting QDPR, not previously detected by Sanger sequencing of QDPR coding regions, exome sequencing, QDPR mRNA PCR, or karyotyping. This case is very good example how genome sequencing is giving an additional diagnostic yield to more common molecular methods. To our knowledge, this is the first report on a large, canonical homozygous pathogenic inversion detected by genome sequencing.

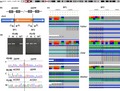

## 1 INTRODUCTION

Tetrahydrobiopterin (BH_4_) is an important cofactor for aromatic amino acid hydroxylases (phenylalanine (Phe), tyrosine (Tyr), and tryptophan hydroxylase) as well as nitric oxide synthase (Opladen, Hoffmann, & Blau, 2012). Disturbances in BH_4_ synthesis or regeneration can lead to disrupted homeostasis of Phe and neurotransmitter synthesis, thus causing hyperphenylalaninemia (HPA); additionally, the lack of neurotransmitters dopamine, noradrenaline, and serotonin may lead to severe cognitive and motor delay (Opladen et al., 2012; Werner, Blau, & Thony, 2011). One of the key enzymes for maintaining BH_4_ supply in the organism is dihydropteridine reductase (DHPR, http://www.chem.qmul.ac.uk/iubmb/enzyme/EC1/6/99/7.html) responsible for BH_4_ regeneration, which is encoded by the quinoid dihydropteridine reductase (*QDPR*) gene (OMIM #612676). Biallelic pathogenic variants in *QDPR* gene lead to BH_4_‐deficient HPA, accompanied with a severe biogenic amines deficiency (OMIM #261630). DHPR deficiency is the second most common form of BH_4_ deficiency (Blau, 2016). According to the online register of BH_4_ deficiencies (http://www.biopku.org/biodef/BIODEF) (Opladen et al., 2012), 303 cases of DHPR deficiency have been recorded. The *QDPR* locus‐specific database PNDdb (http://www.biopku.org/home/pnddb.asp) tabulates information of 85 disease‐causing *QDPR* variants, 50 of them missense, 8 nonsense, 8 small deletions, 7 splice variants, 6 small insertions/duplication, 4 indels, 1 large deletion, and 1 synonymous variant. One intronic variant resulting in cryptic splice site activation has been reported in a patient with DHPR deficiency (Ikeda et al., 1997).

## 2 CASE REPORT

Here we report a case where DHPR deficiency was diagnosed in 1992 by analyzing neurotransmitter and pterin metabolites and confirmed by measuring DHPR activity. The DNA variant causing this phenotype remained unsolved until now, despite repeated targeted sequencing of the *QDPR* gene coding regions and exome sequencing (ES).

The proband was born as the first child of unrelated Estonian parents following a normal pregnancy with birth weight 3,365 g, length 48 cm, and Apgar scores of 8 and 9 at 1 and 5 min, respectively. Since the first week of life, the proband's mother noticed muscular rigidity and lack of eye contact. Later permanent constipation, and contiguous periods of crying with only brief spontaneous laughter was noted. At the age of 6 months she was hospitalized for further investigations of developmental delay and spasticity. On metabolic screening, HPA (plasma Phe = 1,179 µmol/L) was detected, and a low‐Phe diet was started assuming that this child had classical phenylketonuria. However, she did not improve clinically and seizures started one month later. Accordingly, a high suspicion of disturbance in BH_4_ metabolism arose. At the age of 8 months, Phe loading test (0.1 g Phe/kg body weight) was performed at Vilnius Children's Hospital, Lithuania, resulting in a sharp increase in Phe level: at 0 min after consumption—Phe 23.4 µmol/L, Tyr 79.9 µmol/L; at 65 min—Phe 629.3 µmol/L, Tyr 71.4 µmol/L; at 100 min—Phe 1,083 µmol/L, Tyr 91.4 µmol/L). Furthermore, BH_4_ loading test (7 mg BH_4_/kg body mass) on the background of increased Phe level was performed at the age of 9 months, resulting in variable changes in plasma Phe and Tyr: 0 hr—Phe 1,400 µmol/L, Tyr 55.8 µmol/L; 4 hr—Phe 923.7 µmol/L Tyr 55.8 µmol/L; 8 hr—Phe 1555.3 µmol/L Tyr 62.8 µmol/L; 24 hr—Phe 892.3 µmol/L Tyr 48.8 µmol/L, indicating possible disturbance in BH_4_ synthesis/regeneration.

At the age of 11 months, the child was reinvestigated for BH_4_ cofactor disorders. BH_4_ loading test, urinary biopterin analysis, and cerebrospinal (CSF) neurotransmitter analyses showed biochemical abnormalities indicating DHPR deficiency. Neopterin in urine was normal: 1.6 mmol/mol creat (normal: 1.1–4.0 mmol/mol creat) and biopterin was significantly increased: 12.3 mmol/mol creat (normal: 0.5–3.0 mmol/mol creat), resulting in elevated percentage of biopterin (88%). BH_4_ loading test (20 mg/kg body weight) resulted in blood Phe reduction from initial 496 µmol/L to 254 µmol/L and, Phe 169 µmol/L, 4 and 8 hr after BH_4_ administration, respectively. Investigation of CSF neurotransmitters showed normal neopterin—18 nmol/L (normal: 9–40 nmol/L) and elevated biopterin—65 nmol/L (normal: 10–50 nmol/L), very low 5‐HIAA—19 nmol/L (normal: 114–336 nmol/L) and HVA—170 nmol/L (normal: 295–932 nmol/L), 5‐MTHF—26 nmol/L (normal: 64–182 nmol/L), all compatible with DHPR deficiency. Details of all biochemical analyses are given in Table 1. The diagnosis was confirmed by the absent DHPR activity in dried blood (<0.5 µU/g Hb; normal: 2.3–3.8). Accordingly, substitution therapy with L‐DOPA/carbidopa, 5‐OH‐tryptophan and folinic acid as well as continuation of the low‐Phe diet and anticonvulsive therapy were introduced.

**Table 1:** Biochemical investigations and treatment of DHPR deficient patient

Age	Phe (B) µmol/L	Neo (U) mmol/mol creat	Bio (U) mmol/mol creat	%Bio	Neo (CSF) nmol/L	Bio (CSF) nmol/L	%Bio	5OH‐IAA (CSF) nmol/L	HVA (CSF) nmol/L	5MTHF (CSF) nmol/L	Phe (CSF) µmol/L	Tyr (CSF) µmol/L	Dopa/ Carbidopa	5‐OH‐Trp	Folinate
4m2w	1,179														
8m	1,400														
10m	496	1.6	12.3	88	18	65	79	19	170	26	74	43			
1y					11	48	82	257	179	91			3 × 15 mg	3 × 25 mg	1 × 25 mg
1y1m					11	35	76	167	226	72			3 × 15 mg	3 × 25 mg	1 × 25 mg
1y1m2w					10	38	80	296	283	66					
1y10m					7	32	89	521	505				3 × 35 mg	3 × 33 mg	1 × 20 mg
2y					7	32	89	303	381	77					
2y6m					11	34	76	239	340	70					

At the age of 18 months, after 8 months on neurotransmitter substitution therapy, CSF analysis of pterins and neurotransmitter metabolites was unremarkable. The child exhibited her highest psychomotor abilities at the age of 30 months, having eye contact, being able to independently grasp toys and insert them into her mouth; to take a kneeling position and perform jerky motions, and laugh at a whirligig. Since the age of 3 years, her skills started to deteriorate in parallel with slowly progressing muscular atrophy. The child died at the age of 8 years despite treatment. At the time of her death, she exhibited profound developmental delay, generalized seizures and almost no acquired skills.

In two of the following pregnancies, prenatal testing was performed using an enzyme assay—DHPR activity measurement (in chorionic biopsy/amniotic fluid and umbilical cord blood)—which showed mildly decreased level of DHPR activity consistent with fetal heterozygosity for a *QDPR* variant. In an effort to identify the causative variants for DHPR deficiency, *QDPR* exons were Sanger sequenced four times by different laboratories in the index patient and her parents, but no potentially pathogenic variants were identified.

## 3 METHODS

### 3.1 Ethics

All activities performed during this study were approved by Research Ethics Committee of the University of Tartu (approval date 21.09.2015 number 251/T‐6) and were strictly in accordance with the Declaration of Helsinki. Informed consent for carrying out research was obtained from the family of the proband.

### 3.2 Exome sequencing

Exome sequencing of the index patient was carried out in the Estonian Genome Centre at the University of Tartu. DNA library was prepared using Nextera Rapid Capture Exome 37 Mb kit (Illumina Inc.) according to the manufacturer's protocols. The HiSeq 2,500 (Illumina Inc.) platform was used for paired‐end 2 × 100 bp sequencing. The bioinformatics data processing made use of BWA (Li & Durbin, 2009), which mapped the reads to the hg19 reference genome, and different Picard (v2.2.2) and Genome Analysis Toolkit (GATK) (v3.5‐0) tools to mark duplicate reads, recalibrate base quality scores. GATK Haplotype Caller v3.5‐0 was used to call variants.

An in‐house variant annotation pipeline was used. Annotations included, but were not limited to reference databases from ExAC (Lek et al., 2016) and 1,000 Genomes Project (Genomes Project et al., 2015), and ClinVar pathogenicity annotations (Landrum et al., 2016), as well as HPO terms (Kohler et al., 2014) and OMIM disorders as gene‐based annotations. Additionally, allele counts from our in‐house database of variants detected among all NGS analyses (panels and ES) performed in our department (654 samples) were annotated to every detected variant.

CNVs were called using CoNIFER software (Krumm et al., 2012). First, reads per thousand bases per million reads sequenced (RPKM) values were calculated for each sample separately. Second, all available samples using the same library preparation kit were joined for CNV calling. CNV detection and plot generation for detected CNVs were carried out subsequently according to CoNIFER guidelines.

### 3.3 mRNA study

Blood for mRNA analysis was collected from the parents and a control, to obtain cDNA from *QDPR* and analyze the integrity of the cDNA by PCR. PCR was performed from the cDNA synthesized from blood extracted RNA (Tempus™ Spin RNA Isolation Kit, incl DNAse treatment) with SuperScript^TM^ III Reverse Transcriptase (Invitrogen) according to the manufacturer's protocol; the first strand cDNA was synthesized using Random Hexamers (Applied Biosystem). The primers (QDPR_Rev GTGACTTTTCTGGCAGGCCCCTCATA and QDPR_For GGAGCTGCGGGAGCCGGGCT) were designed from UTR regions of the transcript (NCBI Reference Sequence: NM_000320.3), thus an alternative (93 bp) exon was included into the PCR products with predicted 809 and 716 bp fragments depending on the presence/absence of alternative exon. Phusion Hot Start II DNA Polymerase (ThermoFisher) was used for the PCR reaction.

### 3.4 Genome sequencing

Genome sequencing (GS) and data processing were performed by the Genomics Platform at the Broad Institute of MIT and Harvard. PCR‐free preparation of sample DNA (350 ng input at >2 ng/µl) is accomplished using Illumina HiSeq X Ten v2 chemistry. Libraries are sequenced to a mean target coverage of >30×. GS data were processed through a pipeline based on Picard, using base quality score recalibration and local realignment at known indels. The BWA aligner was used for mapping reads to the human genome build 38. Single‐nucleotide variants (SNVs) and insertions/deletions (indels) are jointly called across all samples using Genome Analysis Toolkit (GATK) HaplotypeCaller package version 3.4. Default filters were applied to SNV and indel calls using the GATK Variant Quality Score Recalibration (VQSR) approach. Annotation was performed using Variant Effect Predictor (VEP). Lastly, the variant call set was uploaded to seqr for collaborative analysis between the Center Mendelian Genomics and investigator.

### 3.5 Chromosome analysis

Karyotype analysis from peripheral blood lymphocytes was performed by using conventional GTG‐banding technique (G‐bands by trypsin using Giemsa; band level 550). Karyotypes were described according to the International System for Human Cytogenetic Nomenclature (ISCN2016), (Gonzalez Garcia & Meza‐Espinoza, ). Both karyotypes were performed at the 550‐band level.

## 4 RESULTS

### 4.1 Exome sequencing

Singleton ES failed to reveal pathogenic variants in QDPR gene or in other genes involved in pterin metabolism. However, ES analysis indicated homozygosity around the QDPR gene locus, as only homozygous variants were detected in 10‐Mb region chr4:7433652‐17817262 (GRCh38).

### 4.2 Genome sequencing

**Figure 1:**
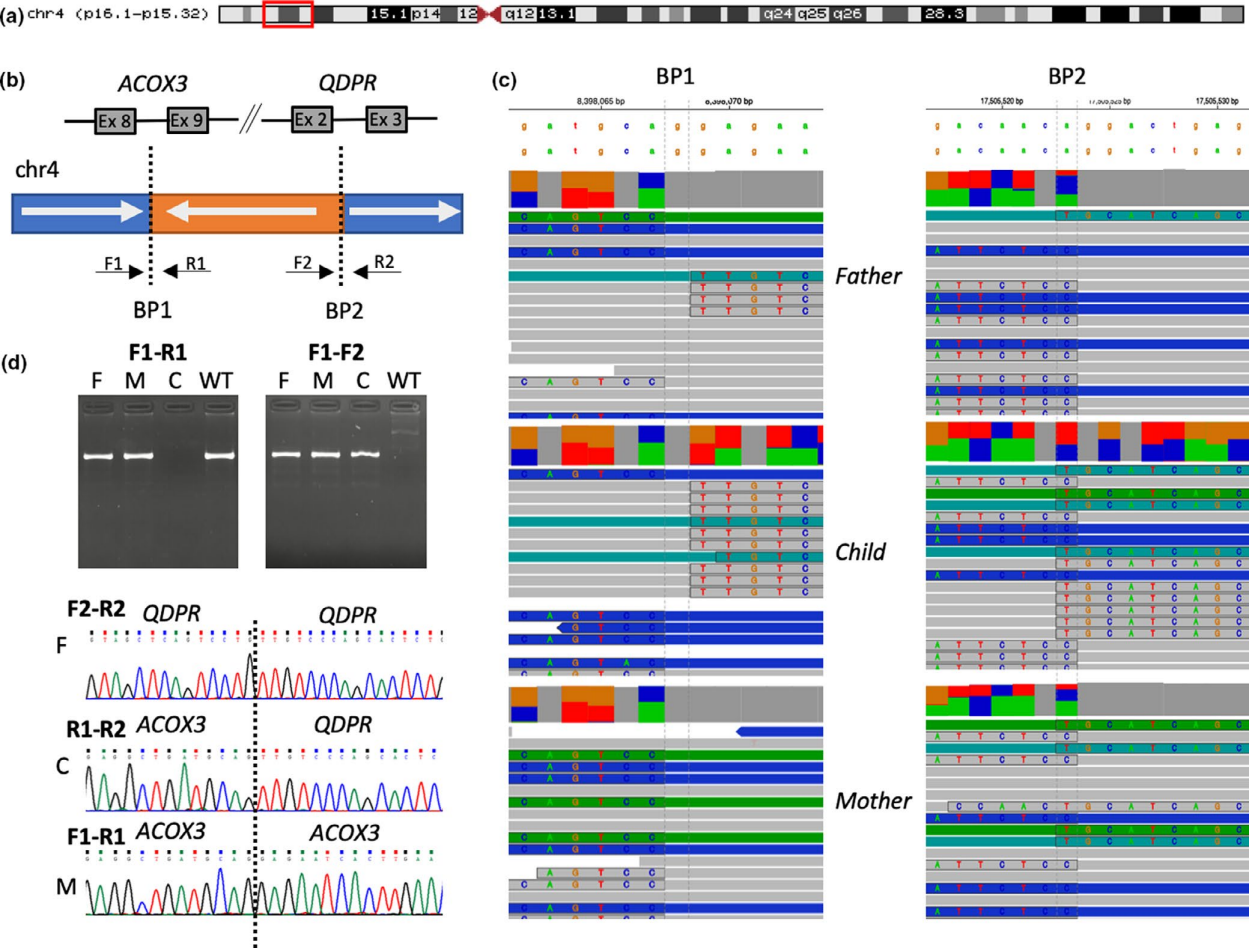
Identification of a new homozygous structural variant in a biochemically diagnosed patient with dihydropteridine reductase (DHPR) deficiency: a 9 Mb inversion between 4p16.1 and 4p15.32. (a) Chromosome 4 ideogram. The described 9‐Mb inversion is marked by the red box. (b) Scheme of the inversion in the context of ACOX3 and QDPR genes. F1, R1, F2, R2 schematically represent the PCR primer design relative to the reference (WT) genome. (c) The aligned sequencing reads around detected breakpoints BP1 and BP2 visualized using The Integrative Genomics Viewer (IGV). Soft‐clipped nucleotides are highlighted and nucleotides shown. (d) Validation studies confirming the variant by PCR using primers F1‐R1 and F1‐F2. C—child, M—mother, F—Father, WT—wild‐type control

Following the initial investigations, predominantly after obtaining the results from ES, the main hypothesis was that the patient may have a homozygous noncoding rearrangement which disrupts *QDPR* expression. To explore this hypothesis, trio GS was performed. Although there were no rare coding variants, concordant with previous studies, two rare homozygous intronic variants in the 3’ region of *QDPR* were detected (c.*119+4759T>C and c.*119+12119G>A, ENST00000513615.5). Both variants were heterozygous in both parents. In the gnomAD database, both variants have an allele frequency below 0.1% with no homozygotes (Karczewski et al., 2019). However, as the functional consequence is hard to predict for intronic single‐nucleotide variants (SNVs), we searched further for other classes of variants. By visual inspecting aligned sequencing reads, a possible structural variant (SV) breakpoint at Chr4(GRCh38):g.17505522 (Figure 1c) was detected, which locates to intron 2 of *QDPR*. The other breakpoint was discovered by paired read mapping and split read analysis, revealing it localized to intron 8 of the *ACOX3* gene (Chr4(GRCh38):g.8398067) (Figure 1c). Thus, a possible 9 megabase (Mb) inversion of 4p was suspected (Figure 1a,b). We also performed *post hoc* analysis using three structural variant callers on GS data: Manta (Chen et al., 2016), DELLY (Rausch et al., 2012), and Smoove (Layer, Chiang, Quinlan, & Hall, 2014). All three SV callers detected the inversion and genotyped it as homozygous in the proband and heterozygous in both parents. To validate the inversion we designed oligonucleotide primers (Figure 1b, Table 2) to perform PCR amplification, and subsequent Sanger validation around the breakpoints. As expected, the proband did not have any PCR product using F1‐R1, and F2‐R2 primer pairs, whereas F1‐F2 and R1‐R2 primer pairs gave PCR product with predicted sequence over both breakpoints (Figure 1d). This 9‐Mb inversion was not observed in 10,782 unrelated genomes with SV calls in gnomAD (Collins et al., 2019); in fact, no inversions within 544kb of *QDPR* were observed in gnomAD, further supporting the rareness and probable pathogenicity of this inversion. Importantly, gnomAD database has 2,297 Estonians in its GS dataset, thus we can conclude that this inversion is very rare among Estonians as well. As gnomAD SV calling pipeline incorporated two of the tools used in this study for SV calling (Manta and DELLY), then it is highly unlikely that the variants are absent from gnomAD database just due to the differences in SV calling algorithms. Homozygosity mapping from GS data was performed using PLINK 1.9 (Chang et al., 2015), and the homozygous stretch encompassing the inversion was confirmed for the region chr4:7452118‐18823503 (GRCh38), and no other homozygous stretches larger than 5 megabases were detected, thus excluding the parental consanguinity. The detected inversion was submitted to ClinVar database (accession number SCV000898485) and to the locus‐specific PNDdb. Data available on request from the authors.

**Table 2:** PCR primers for validation studies (Figure 1b, 1d)

*ACOX3*	
F1	TGCATGAAGACAGTGGAATCA
R1	AGGAATCACAGTCTCGTTGT
*QDPR*	
F2	TCATGAAACTGGGGAAAGAGGT
R2	AGTTTCGCTTGTCTCCCAGG

### 4.3 Chromosome analysis

**Figure 2:**
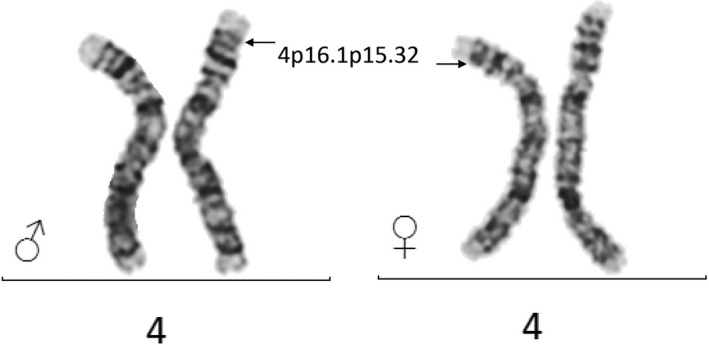
Karyotype analysis from peripheral blood lymphocytes of the parents of the DHPR deficient proband with 9 Mb inversion does not reveal observable pattern abnormality

After finding the large inversion, we investigated whether the inversion could be detected with a regular chromosomal microscopy analysis (karyotyping with G bands) in both heterozygous parents. Our result showed that the inversion of 4p16.1‐p15.32 is not detectable by GTG‐banding techniques, as inversion points 4p16.1 and 4p15.32 form symmetrical pattern around the band 4p16.2 (Figure 2). Accordingly, in spite of the ca 10 MB size of the inversion, the inversion is not detectable by G‐banding.

### 4.4 mRNA analysis

**Figure 3:**
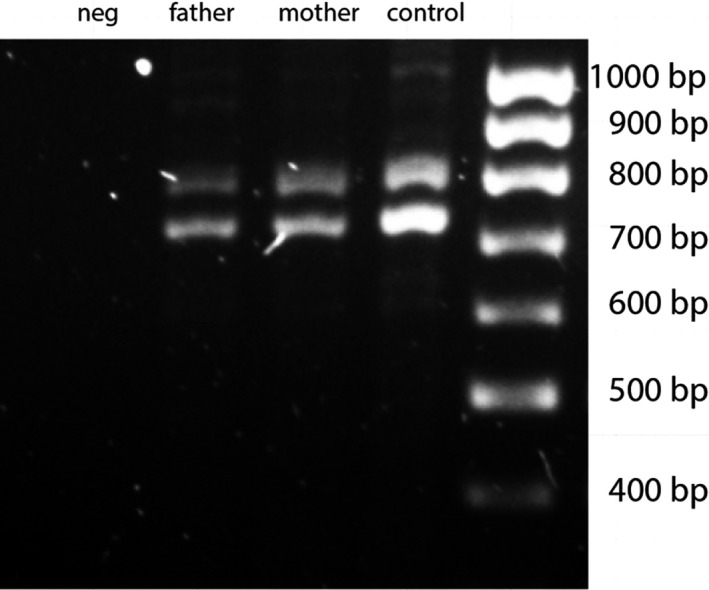
PCR analysis of *QDPR* from the cDNA obtained from peripheral blood mRNA of the parents of the proband and control. No products of abnormal length can be observed, predicted 809 and 716 bp fragments are present. neg: includes all ingredients and primers without cDNA; marker: SolisBiodyne 100 bp DNA ladder

For the identification of variants in *QDPR*, we performed an mRNA studies to analyze the integrity of the cDNA by PCR in heterozygous parental samples (no RNA samples were stored from the proband). This analysis did not reveal any different patterns between the parents of the proband and the control, thus indicating the lack of possible mRNA products with abnormal length (Figure 3). Despite the qualitative essence of the performed reactions, a hint for decreased quantity of obtained *QDPR* cDNA could be retrieved from the visually observed lower intensity of the PCR products of the parents compared to the ones from the control.

## 5 DISCUSSION

This case illustrates both the advantages of GS and the clinical importance of SVs in disease‐associated genes including the ones where SVs have not been previously implicated as a disease mechanism such as *QDPR*. In this family, GS was able to resolve a 27‐year‐long diagnostic odyssey, and now provides the possibility to adequately counsel and test family members at risk for being carriers for this disorder. As the parents are nonconsanguineous, but carry the same inversion and haplotype, this variant may not be unique to this family, furthering the importance of this novel discovery.

SVs, including inversions, are well recognized as a disease mechanism (Collins et al., 2017), but balanced SVs in particular remain difficult to detect using common molecular DNA variant detection assays that typically only focus on coding regions of the genome. Heterozygous and hemizygous inversions are known to cause many different disorders. For example, the most common variant causing severe hemophilia A is a hemizygous inversion in intron 22 of the *F8* gene (Lakich, Kazazian, Antonarakis, & Gitschier, 1993). In addition to small intragenic inversions, some very large inversion detectable by karyotyping has been reported (since the discovery of banding techniques for karyotyping). For example, an early study described a phenotype‐causing homozygous paracentric inversion of chromosome 12 inherited from consanguineous parents (Price, Roberts, & Laurence, 1987). However, karyotyping does not allow for the identification of exact breakpoints and thus the discovery of disrupted genes. This limitation may be overcome by laborious breakpoint searching using PCR and Sanger sequencing, as seen in a recently published homozygous inversion causing Phosphoglucomutase 1 deficiency (Yokoi et al., 2019). A systematic approach was taken by Redin et al. in a large study aiming to map breakpoints of balanced chromosomal aberrations in individuals with congenital anomalies (Redin et al., 2017) using a lower‐resolution “long‐insert” (also known as “mate‐pair”) GS technology. The authors demonstrated that in third of the patients carrying cytogenetically balanced aberration, breakpoint mapping revealed disruption of a gene probably contributing to the phenotype. While GS has facilitated detections of some disease causing inversions also in the absence of prior suspicion to chromosomal rearrangement (Grigelioniene et al., 2017; Kim et al., 2015), these variants remain rare and probably under detected as GS is still much less available than ES in both research and clinical settings. Moreover, even if using GS, the short‐read sequencing technologies like Illumina sequencing used in this study, may have reduced sensitivity in SV detection due to repetitive sequences around breakpoints or more complex nature of some SVs. Thus, long‐read sequencing approaches like single molecule real‐time (SMRT) sequencing by Pacific Biosciences or nanopore sequencing by Oxford Nanopore Technologies Inc. may be needed to identify some SVs. To our knowledge, this is the first report on a large, canonical (rather than complex) homozygous pathogenic inversion detected by GS. In addition to simple inversions, GS can be used to resolve more complex SVs, where inversions may be accompanied by deletions or duplications (Sanchis‐Juan et al., 2018). Accurate detection of SVs also requires adequate variant calling software. Our post hoc analysis of three SV_calling tools demonstrated that all three tools identified the inversion correctly, illustrating that many currently available algorithms are capable of detecting disease causing inversions.

Interestingly, the second breakpoint of our patient's inversion was mapped into the intronic area between exons 8 and 9 of the *ACOX3* gene encoding pristanoyl‐CoA oxidase (OMIM *603402). This enzyme has been shown to be involved in the degradation of the branched‐chain fatty acids (Ferdinandusse et al., 2018; Vanhooren, Marynen, Mannaerts, & Van Veldhoven, 1997), but its deficiency is probably compensated for by other peroxisomal acyl‐CoA oxidases and we do not feel that this gene disruption is clinically manifesting in our patient.

From a clinical perspective, the current report presents an additional strategy for finding a molecular diagnoses, if biochemical features are suggestive of a disorder and conventional or even the most up‐to‐date methods fail to identify the causative variant. Although high‐throughput targeted sequencing has been shown to be very effective in solving BH_4_‐deficient HPAs (Trujillano et al., 2014), our case reiterates the additional benefits associated with GS. This inversion maintained all of the exons of *QDPR* intact, as the breakpoint occurred deep inside of an intron, thus presented completely normal results from PCR and Sanger sequencing as well as ES. Obviously, the presence of a breakpoint inside a gene eliminates the possibility of synthesizing normal functional mRNA and respective protein. We also demonstrate the inability of standard cytogenetic methods to reveal this inversion. After mapping the breakpoints in the genome of the index patient, further diagnostic testing in the family can easily be performed by simple PCR reactions like presented in Figure 1d, and/or Sanger sequencing, enabling significantly faster analysis and consultation, especially for prenatal diagnosis.

## CONFLICT OF INTEREST

The authors declare no conflict of interest.

## References

[mgg31154-bib-0001] Blau, N. (2016). Genetics of phenylketonuria: Then and now. Human Mutation, 37(6), 508–515. 10.1002/humu.22980 26919687

[mgg31154-bib-0002] Chang, C. C. , Chow, C. C. , Tellier, L. C. , Vattikuti, S. , Purcell, S. M. , & Lee, J. J. (2015). Second‐generation PLINK: Rising to the challenge of larger and richer datasets. Gigascience, 4, 7 10.1186/s13742-015-0047-8 25722852 PMC4342193

[mgg31154-bib-0003] Chen, X. , Schulz‐Trieglaff, O. , Shaw, R. , Barnes, B. , Schlesinger, F. , Källberg, M. , … Saunders, C. T. (2016). Manta: Rapid detection of structural variants and indels for germline and cancer sequencing applications. Bioinformatics, 32(8), 1220–1222. 10.1093/bioinformatics/btv710 26647377

[mgg31154-bib-0004] Collins, R. L. , Brand, H. , Karczewski, K. J. , Zhao, X. , Alföldi, J. , Khera, A. V. , Talkowski, M. E. (2019). An open resource of structural variation for medical and population genetics. bioRxiv. 10.1101/578674

[mgg31154-bib-0005] Collins, R. L. , Brand, H. , Redin, C. E. , Hanscom, C. , Antolik, C. , Stone, M. R. , … Talkowski, M. E. (2017). Defining the diverse spectrum of inversions, complex structural variation, and chromothripsis in the morbid human genome. Genome Biology, 18(1), 36 10.1186/s13059-017-1158-6 28260531 PMC5338099

[mgg31154-bib-0006] Ferdinandusse, S. , Denis, S. , van Roermund, C. W. T. , Preece, M. A. , Koster, J. , Ebberink, M. S. , … Wanders, R. J. A. (2018). A novel case of ACOX2 deficiency leads to recognition of a third human peroxisomal acyl‐CoA oxidase. Biochimica et Biophysica Acta (BBA) ‐ Molecular Basis of Disease, 1864(3), 952–958. 10.1016/j.bbadis.2017.12.032 29287774

[mgg31154-bib-0007] Genomes Project , Auton, A. , Brooks, L. D. , Durbin, R. M. , Garrison, E. P. , Kang, H. M. , … Abecasis, G. R. (2015). A global reference for human genetic variation. Nature, 526(7571), 68–74. 10.1038/nature15393 26432245 PMC4750478

[mgg31154-bib-0008] Gonzalez Garcia, J. R. , & Meza‐Espinoza, J. P. Use of the International System for Human Cytogenetic Nomenclature (ISCN). Blood, 108(12), 3952–3953. 10.1182/blood-2006-06-031351 17114573

[mgg31154-bib-0009] Grigelioniene, G. , Nevalainen, P. I. , Reyes, M. , Thiele, S. , Tafaj, O. , Molinaro, A. , … Juppner, H. (2017). A large inversion involving GNAS exon A/B and all exons encoding gsalpha is associated with autosomal dominant pseudohypoparathyroidism type Ib (PHP1B). Journal of Bone and Mineral Research, 32(4), 776–783. 10.1002/jbmr.3083 28084650 PMC5395346

[mgg31154-bib-0010] Ikeda, H. , Matsubara, Y. , Mikami, H. , Kure, S. , Owada, M. , Gough, T. , … Narisawa, K. (1997). Molecular analysis of dihydropteridine reductase deficiency: Identification of two novel mutations in Japanese patients. Human Genetics, 100(5–6), 637–642. 10.1007/s004390050566 9341885

[mgg31154-bib-0011] Karczewski, K. J. , Francioli, L. C. , Tiao, G. , Cummings, B. B. , Alföldi, J. , Wang, Q. , MacArthur, D. G. (2019). Variation across 141,456 human exomes and genomes reveals the spectrum of loss‐of‐function intolerance across human protein‐coding genes. bioRxiv. 10.1101/531210

[mgg31154-bib-0012] Kim, J. , Won, H. H. , Kim, Y. , Choi, J. R. , Yu, N. , & Lee, K. A. (2015). Breakpoint mapping by whole genome sequencing identifies PTH2R gene disruption in a patient with midline craniosynostosis and a de novo balanced chromosomal rearrangement. Journal of Medical Genetics, 52(10), 706–709. 10.1136/jmedgenet-2015-103001 26044810 PMC4621369

[mgg31154-bib-0013] Köhler, S. , Doelken, S. C. , Mungall, C. J. , Bauer, S. , Firth, H. V. , Bailleul‐Forestier, I. , … Robinson, P. N. (2014). The Human Phenotype Ontology project: Linking molecular biology and disease through phenotype data. Nucleic Acids Research, 42(D1), D966–D974. 10.1093/nar/gkt1026 24217912 PMC3965098

[mgg31154-bib-0014] Krumm, N. , Sudmant, P. H. , Ko, A. , O'Roak, B. J. , Malig, M. , Coe, B. P. , … Eichler, E. E. (2012). Copy number variation detection and genotyping from exome sequence data. Genome Research, 22(8), 1525–1532. 10.1101/gr.138115.112 22585873 PMC3409265

[mgg31154-bib-0015] Lakich, D. , Kazazian, H. H. Jr , Antonarakis, S. E. , & Gitschier, J. (1993). Inversions disrupting the factor VIII gene are a common cause of severe haemophilia A. Nature Genetics, 5(3), 236–241. 10.1038/ng1193-236 8275087

[mgg31154-bib-0016] Landrum, M. J. , Lee, J. M. , Benson, M. , Brown, G. , Chao, C. , Chitipiralla, S. , … Maglott, D. R. (2016). ClinVar: Public archive of interpretations of clinically relevant variants. Nucleic Acids Research, 44(D1), D862–868. 10.1093/nar/gkv1222 26582918 PMC4702865

[mgg31154-bib-0017] Layer, R. M. , Chiang, C. , Quinlan, A. R. , & Hall, I. M. (2014). LUMPY: A probabilistic framework for structural variant discovery. Genome Biology, 15(6), R84 10.1186/gb-2014-15-6-r84 24970577 PMC4197822

[mgg31154-bib-0018] Lek, M. , Karczewski, K. J. , Minikel, E. V. , Samocha, K. E. , Banks, E. , Fennell, T. , … MacArthur, D. G. (2016). Analysis of protein‐coding genetic variation in 60,706 humans. Nature, 536(7616), 285–291. 10.1038/nature19057 27535533 PMC5018207

[mgg31154-bib-0019] Li, H. , & Durbin, R. (2009). Fast and accurate short read alignment with Burrows‐Wheeler transform. Bioinformatics, 25(14), 1754–1760. 10.1093/bioinformatics/btp324 19451168 PMC2705234

[mgg31154-bib-0020] Opladen, T. , Hoffmann, G. F. , & Blau, N. (2012). An international survey of patients with tetrahydrobiopterin deficiencies presenting with hyperphenylalaninaemia. Journal of Inherited Metabolic Disease, 35(6), 963–973. 10.1007/s10545-012-9506-x 22729819

[mgg31154-bib-0021] Price, H. A. , Roberts, S. H. , & Laurence, K. M. (1987). Homozygous paracentric inversion 12 in a mentally retarded boy: A case report and review of the literature. Human Genetics, 75(2), 101–108. 10.1007/BF00591068 3546078

[mgg31154-bib-0022] Rausch, T. , Zichner, T. , Schlattl, A. , Stutz, A. M. , Benes, V. , & Korbel, J. O. (2012). DELLY: Structural variant discovery by integrated paired‐end and split‐read analysis. Bioinformatics, 28(18), i333–i339. 10.1093/bioinformatics/bts378 22962449 PMC3436805

[mgg31154-bib-0023] Redin, C. , Brand, H. , Collins, R. L. , Kammin, T. , Mitchell, E. , Hodge, J. C. , … Talkowski, M. E. (2017). The genomic landscape of balanced cytogenetic abnormalities associated with human congenital anomalies. Nature Genetics, 49(1), 36–45. 10.1038/ng.3720 27841880 PMC5307971

[mgg31154-bib-0024] Sanchis‐Juan, A. , Stephens, J. , French, C. E. , Gleadall, N. , Mégy, K. , Penkett, C. , … Carss, K. J. (2018). Complex structural variants in Mendelian disorders: Identification and breakpoint resolution using short‐ and long‐read genome sequencing. Genome Medicine, 10(1), 95 10.1186/s13073-018-0606-6 30526634 PMC6286558

[mgg31154-bib-0025] Trujillano, D. , Perez, B. , González, J. , Tornador, C. , Navarrete, R. , Escaramis, G. , … Estivill, X. (2014). Accurate molecular diagnosis of phenylketonuria and tetrahydrobiopterin‐deficient hyperphenylalaninemias using high‐throughput targeted sequencing. European Journal of Human Genetics, 22(4), 528–534. 10.1038/ejhg.2013.175 23942198 PMC3953903

[mgg31154-bib-0026] Vanhooren, J. C. , Marynen, P. , Mannaerts, G. P. , & Van Veldhoven, P. P. (1997). Evidence for the existence of a pristanoyl‐CoA oxidase gene in man. The Biochemical Journal, 325(Pt 3), 593–599. 10.1042/bj3250593 9271077 PMC1218600

[mgg31154-bib-0027] Werner, E. R. , Blau, N. , & Thony, B. (2011). Tetrahydrobiopterin: Biochemistry and pathophysiology. The Biochemical Journal, 438(3), 397–414. 10.1042/BJ20110293 21867484

[mgg31154-bib-0028] Yokoi, K. , Nakajima, Y. , Ohye, T. , Inagaki, H. , Wada, Y. , Fukuda, T. , … Kurahashi, H. (2019). Disruption of the responsible gene in a phosphoglucomutase 1 deficiency patient by homozygous chromosomal inversion. JIMD Reports, 43, 85–90. 10.1007/8904_2018_108 29752652 PMC6323009

